# Central nervous system involvement in patients with acute myeloid leukemia

**DOI:** 10.3906/sag-2103-127

**Published:** 2021-10-21

**Authors:** Hatime Arzu YAŞAR, Olgu Erkin ÇINAR, Nur YAZDALI KÖYLÜ, İbrahim BARIŞTA, Hakan GÖKER, Yahya BÜYÜKAŞIK

**Affiliations:** 1 Department of Internal Medicine, Faculty of Medicine, Hacettepe University, Ankara Turkey; 2 Division of Hematology, Department of Internal Medicine, Faculty of Medicine, Hacettepe University, Ankara Turkey; 3 Division of Medical Oncology, Department of Internal Medicine, Faculty of Medicine, Hacettepe University, Ankara Turkey

**Keywords:** Acute myeloid leukemia, central nervous system involvement, risk factors

## Abstract

**Background/aim:**

To evaluate the incidence, clinical features, risk factors, and prognosis of central nervous system (CNS) involvement in patients with acute myeloid leukemia (AML).

**Materials and methods:**

All AML patients who were admitted to Hacettepe University hospital between 2000 and 2021 were evaluated. The medical records of 548 AML cases were retrospectively analyzed.

**Results:**

The frequency of CNS involvement was 2.4% (n = 13) at diagnosis and 4.6% (n = 25) at diagnosis or during follow-up. Parenchymal involvement was seen in 5 patients, leptomeningeal involvement was seen in 11 patients. Three patients had both leptomeningeal and parenchymal involvements, and 6 patients had optic nerve or ocular involvement. In univariate analysis, younger age and extramedullary involvement at diagnosis were associated with CNS disease at diagnosis, and extramedullary involvement at diagnosis was associated with CNS disease during follow-up. In multivariate analysis; younger age and extramedullary involvement at diagnosis were associated with CNS disease at diagnosis and during follow-up respectively. Median overall survival was 5.4 months in patients with CNS disease at diagnosis and 16.9 months in patients with CNS disease during follow-up and 16.2 months in patients with no CNS disease.

**Conclusion:**

CNS disease is a rare complication of AML. Younger age and extramedullary involvement at diagnosis are risk factors for CNS involvement.

## 1. Introduction

Acute myeloid leukemia (AML) was the most frequent acute leukemia in adult patients. The incidence is around 3–5 per 100,000 [1]. The central nervous system (CNS) involvement in patients with AML is rare and not identified certainly. Therefore, baseline screening is not recommended for the patients without symptoms [2,3]. Clinically symptomatic CNS involvement is not common after treatment. The factors related to CNS involvement are reported as monocytic component predominant AML, systemic relapse of acute promyelocytic leukemia (APL), presence of inversion 16, chromosome 11 anomalies, hyperleukocytosis, lactate dehydrogenase (LDH) elevation, and *FLT3*–internal tandem duplication (ITD) mutations [4–9]. Leukemic cells expressing CD56 adhesion molecule on cell surface were also investigated as a risk factor for CNS involvement [10,11]. It was found that CNS involvement in patients with AML usually presents as leptomeningeal involvement, but it may also rarely present as a mass in the brain parenchyma in acute myelomonocytic leukemia (French-American-British, FAB, AML-M4 subtype) patients with inv(16)(p13q22) or del(16)(q22) inversion 16 (+) AML [5].

In this study, we aimed to determine the incidence, type, risk factors, and prognosis of CNS involvement in patients with AML.

## 2. Materials and methods

All AML patients who were admitted to Hacettepe University Faculty of Medicine Department of Hematology between 2000 and 2021 were evaluated. Clinical and laboratory data were obtained from patient files and the hospital electronic database. The diagnosis of AML and morphological classification were done using valid international criteria at the time of presentation using bone marrow and peripheral blood microscopic evaluations and flow cytometric analysis. Organomegaly, lymphadenopathy, gum hypertrophy, and histologically documented any tissue involvement were evaluated as extramedullary involvement. The diagnosis of CNS involvement was done by radiological evaluations in patients with suspicious neurological symptoms. Cerebrospinal fluid analysis, biopsies, and ophthalmological examinations were also performed when clinically indicated.

Treatment of non- APL AML in patients who were considered suitable for intensive therapy was done by a 3+7 regimen containing an anthracycline (idarubicin, mitoxantrone, or daunorubicin) for 3 days and standard-dose cytarabine for 7 days. Two to 4 cycles of high-dose cytarabine regimens were applied for consolidation [12]. APL patients were treated with all-trans-retinoic acid and anthracycline-based induction and consolidation (generally 3 cycles) regimens [13]. Routine CNS disease evaluations and prophylaxis were not done in any type of disease. Allogeneic stem cell transplantation was aimed in patients without favorable AML subtypes (i.e. APL, CBF-type, and during last few years mutated NPM1/without FLT3-ITD, and biallelic CEBPA mutation).

The Ethics Committee of Hacettepe University Faculty of Medicine approved the study protocol (date: 30.04.14, registration number: GO 14/234).

### 2.1. Statistical analyzes

SPSS version 17.0 (SPSS Inc, Chicago, IL, USA) was used for statistical analyzes. Categorical data were expressed as number (and/or ratio). Continuous data were shown as median (range). Chi-square test was used to determine whether there was any difference between the CNS involvement subgroups (i.e. no involvement, involvement at diagnosis, involvement at relapse) in terms of categorical variables. In the statistical comparison of continuous variables between the CNS involvement groups, Kruskal–Wallis H test was used. Univariate and multivariate logistic regression analyzes were performed to determine possible parameters at diagnosis that may be related to CNS involvement at the time of diagnosis, at relapse, or anytime. Eastern Cooperative Oncology Group (ECOG) performance score was excluded from these analyzes, because the presence of CNS involvement at the time of diagnosis is expected to impair performance status. 

Overall survival was used for prognostic evaluation. The time from diagnosis to death for any reason was calculated. The patients who were still alive when they were last seen were censored on this date. Overall survival was calculated according to the Kaplan–Meier method. The comparison between survival curves was done by log-rank test.

P values < 0.05 were considered statistically significant. The parameters with at least borderline significance (p < 0.1) in univariate logistic regression analysis were included in the multivariate logistic regression analysis.

## 3. Results

### 3.1. Patients

Five hundred forty-eight patients were included in the study. A total of 58% of them were male (n = 318) and 42% were female (n = 230). The median age was 50.9 (18.3–88,1.0). The median follow-up duration of the surviving patients was 32.3 (0.03–358.7) months. Patient characteristics are presented in Table 1.

**Table 1:** Characteristics of the patient groups.

	CNS involvement				p value
	At diagnosis(N: 13)	Only duringrelapse (N: 12)	Anytime(N:25)	Absent(N: 523)	
Sex (F/M)	4/9	6/6	10/15	220/303	0.61
Age (median, range)	31.4 (18.9–49.5)	48.5 (25.6–72.7)	40.2 (18.9–72.7)	51.9 (18.3–88.1)	<0.001
Age (<65 vs older)	13/0	11/1	24/1	391/132	0.016
ECOG (0–1 vs. 2–4)	7/6	8/4	15/10	419/93	0.018
De novo/secondary AML	13/0	9/3	22/3	445/78	0.19
AML M4-5 vs other	2/5	4/5	6/10	76/221	0.44
White blood cell (median, range)	10600 (2300–231600)	39850 (700–98700)	18900 (700–231600)	7850 (200–321300)	0.19
White blood cell (>30x109/L) (yes/no)	3/10	6/4	9/14	129/335	0.075
Lactate dehydrogenase (median, range)	632 (236–7222)	552 (277–2755)	619 (236–7222)	490 (65–8490)	0.43
Lactate dehydrogenase (>1000 IU/L) (yes/no)	3/6	3/5	6/11	71/243	0.47
B2-microglobulin (median, range)	1950 (1402–2705)	1584 (430–1733)	1584 (430–2705)	2243 (818–8372)	0.12
Other extramedullary involvement (yes/no)	7/5	9/2	16/7	104/360	<0.001
ELN* genetic risk group(Favorable/Intermediate/High)	2/3/3	3/3/4	5/6/7	81/148/55	0.35
CBF**-type vs other	1/6	1/7	2/13	27/195	0.98
APL@ vs others	1/5	1/5	2/10	33/153	0.99
Duration between diagnosis-CNS involvement (months)	0 (0–0.47)	8.6 (0.8–49.2)	0.46 (0–49.2)	NA	
Patern of involvement(LM@@, LM&P#, P, Optic nerve/Eye)	5, 2, 1, 4	6, 1, 4, 1	11, 3, 5, 5	NA	0.25
Method of diagnosis (Clinical&radiological/histopathological)	8/4	9/3	17/7	NA	0.65
Systemic high-dose## chemotherapy/intratecal chemotherapy/ CNS radiotherapy	7/8/2	1/5/1	8/13/3	NA	
Allogeneic stem cell transplant(yes/no)§	1/12	5/7**	6/19	131/390	0.14

The sum of the values in the cells may be less than the total number due to missing values *European LeukemiaNet, **core-binding factor, @acute promyelocytic leukemia, @@leptomeningeal, #parenchymal, ##Higher than 1g/m2 cytarabine and/or 500 mg/m2 methotrexate, §before CNS involvement in 4 cases, NA: not applicable.

### 3.2. Incidence and type of CNS involvement 

The incidence of CNS involvement in patients with AML was 2.4% (n = 13) at the time of diagnosis. Twelve cases developed CNS disease during follow-up in spite of no involvement at the time of diagnosis. So, the frequency of CNS involvement at the time of diagnosis or follow-up was 4.6% (n = 25).

Parenchymal involvement was seen in 5 patients, leptomeningeal involvement was seen in 11 patients. Three patients had both leptomeningeal and parenchymal involvement, and 6 patients had optic nerve or ocular involvement. Cranial palsy, mental alteration, head and neck pain, seizure, paralysis, blurred vision, and loss of balance were seen as symptoms in patients with CNS involvement. 

### 3.3. Risk factors for CNS involvement

Univariate and multivariate analyzes for CNS involvement at diagnosis, at relapse and anytime are summarized in Table 2. In the univariate analysis, age, white blood cell, lactate dehydrogenase (LDH), β2 microglobulin, extramedullary involvement, number and location of involvement, AML subtypes, and flow cytometry results were evaluated. Age was associated with CNS involvement at the time of diagnosis in univariate (p = 0.001) and multivariate analyzes (p = 0.006). Extramedullary involvement at diagnosis was related to CNS involvement during follow-up in univariate (p < 0.001) and multivariate analyzes (p = 0.001). Both age and extramedullary involvement were related to CNS involvement at any time in univariate(p = 0.002 and p = 0.001, respectively) and multivariate (p = 0.002 and p = 0.001, respectively) analyzes.

**Table 2:** Univariate and multivariate analyzes for CNS involvement at diagnosis, at relapse and anytime.

	Univariate logistic regression*		Multivariate logistic regression**	
	Exp(B) (95% CI#)	p value	Exp(B)	p value
Risk factors for CNS involvement at diagnosis				
Age	1.089 (1.037–1.143)	0.001	1.091 (1.025–1.162)	0.006
Lactate dehydrogenase	1 (1–1.001)	0.075		
Other extramedullary involvement	4.48 (1.39–14.4)	0.012		
Risk factors for CNS involvement at relapse				
>30x109/L white blood cell	3.127 (0.938–10.421)	0.063		
Other extramedullary involvement	16.591 (3.581–76.856)	<0.001	12.934 (2.712–61.681)	0.001
Risk factors for CNS involvement anytime				
Age	1.044 (1.016–1.073)	0.002	1.049 (1.017–1.082)	0.002
Other extramedullary involvement	7.912 (3.170–19.747)	<0.001	6.263 (2.428–16.156)	<0.001

*Parameters selected for multivariate analysis (p < 0.1) are presented. ** Significant parameters (p < 0.05) are presented. # confidence interval.

### 3.4. The prognosis

The median overall survival duration of patients with CNS involvement at diagnosis (5.4 months [0–13.6; 95% confidence interval, CI]) was shorter than those without CNS involvement (16.2 months [10.4–22; 95% CI]) or CNS involvement during follow-up (16.9 months [0–62.4; 95% CI]). No statistical significance was observed (p = 0.5) probably due to the low patient number (Figure). 

**Figure:**
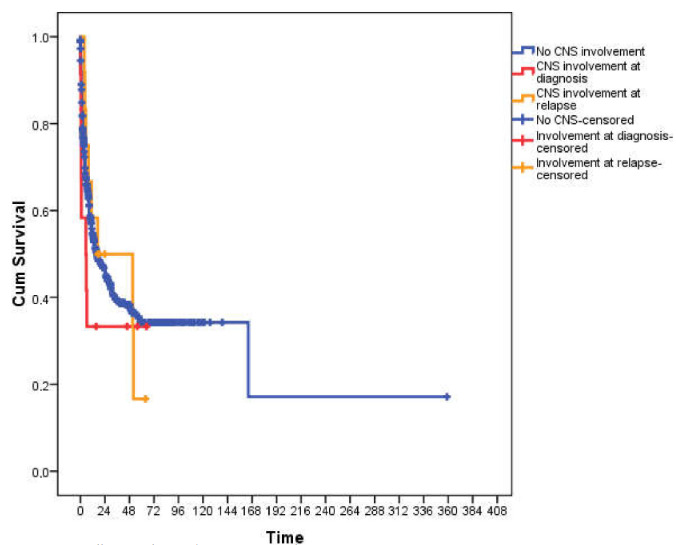
Overall survival according to CNS involvement status.

## 4. Discussion

In the literature, data about CNS involvement in adult patients with AML is rare. The CNS involvement data generally comes from studies involving pediatric AML patients [14,15]. In our study, CNS involvement in AML was 2.4% at the time of diagnosis, and 4.6% throughout the disease course. Abbot et al. and Felix et al. showed CNS involvement in 29% and 16% of patients at the time of diagnosis in pediatric population, respectively [16,14]. These CNS involvement rates were quite higher than adult population. Del Principe et al. showed CNS involvement with routine CNS evaluation regardless of clinical symptoms in 11% of adult patients at the time of diagnosis [17]. In other studies, CNS involvement rates were between 0.6% and 1.8% in patients with AML at the time of diagnosis. CNS involvement rates were between 2.9% and 5.1% throughout the disease [8,7,18,19,9]. Del Principe et al. reported that FAB M4-M5 type and high LDH levels were associated with CNS involvement at the time of diagnosis [17] Cheng et al. showed that high white blood cell counts and inversion 16 were associated with CNS involvement at the time of diagnosis. Alakel et al. showed that complex karyotypes, high LDH levels, FAB M5, and FLT3 mutation were related to CNS involvement at the time of diagnosis [8]. Rozovski et al reported that young age, African-American ethnicity and high LDH levels were risk factors for CNS involvement. Holmes et al. [5] and Shihadeh et al. [6] reported a higher incidence of CNS involvement in APL and FAB M4-M5 AML respectively compared to other AML types. Some studies have shown that high white blood cell count, peripheral eosinophilia, high LDH level, male sex, age <50 were risk factors for CNS involvement at the time of diagnosis [20,18,21]. Jabbour et al. showed that age, high LDH level, and FLT 3 mutation were independent risk factors for CNS involvement in multivariate regression model. In our study, younger age was associated with CNS involvement at the time of diagnosis. We found that extramedullary involvement at diagnosis was related to CNS involvement during follow-up. Age and extramedullary involvement were related to CNS involvement at any time. 

Although CNS involvement at diagnosis was not a prognostic factor in pediatric patients with AML [15,16], it was associated with poor prognosis in adult AML patients [20,22]. Castagnola et al. reported that median overall survival after CNS involvement was 2 months (1–5 months) [2]. Del Principe et al. and Alakel et al. showed that patients with CNS involvement at the time of diagnosis had shorter overall survival [17-8]. In our study, the median overall survival in patients with CNS involvement at the time of diagnosis was shorter than patients who did not experience this complication as similar to the other studies. There was no statistically significant difference between the two groups probably due to low number of patients with CNS disease. 

Our study has the advantage of a high total patient number, diagnosed during two decades. We are aware that it has some limitations inherent to retrospective studies, such as some missing data. 

## 5. Conclusion

CNS involvement occurs rarely (4.6%) in AML. Younger age and extramedullary involvement at diagnosis are risk factors for CNS involvement.
